# Multiple Cystic Kidney

**Published:** 1915-12

**Authors:** Wm. Stanton

**Affiliations:** Webster, N. Y.


### Multiple Cystic Kidney.

### Report of a Case.

By WM. STANTON, M. D., Webster, N. Y.

Mrs. L. M. G. age 24, from childhood has passed a large amount of urine, but being apparently in perfect health never gave it serious thought or consulted a physician, considering it normal for her. The diseases of childhood passed over her lightly, and she was never seriously ill until, while at her summer home during college vacation in 1912, when she was suddenly attacked with a violent headache and vomiting, followed by slight stupor. The diagnosis at the time was acute indigestion, and her recovery required some two or three weeks before she felt that she was again in perfect health. She returned to school, and in the class of 1913 completed a brilliant college course, leading her classes and taking an active part in the college athletics, including rowing and swimming, at both of which she was more than ordinarily skilful. She then taught one year in the Rochester School for the Deaf, where she was considered exceptionally promising in ability. In September, 1914, she married but was never pregnant.

During the night of February 26, 1915, she suffered an attack similar to that in 1912, except that there was very little stupor and that there was also severe pain in the right lumbar region, which led to the discovery of a tumor lying below the liver, and which was evidently the seat of the abdominal pain. Her attending physician offered no positive diagnosis, but advised her to go to her parents, consult their physician and a surgeon as soon as her condition would allow, believing an operation advisable if not necessary.

I first saw her March 4 upon her arrival at her father's home, and the same day took her to the Park Avenue Hospital in Rochester, where she was seen in consultation by Dr. W. B. Jones. The only abnormalities discoverable at this time was a trace of albumin, polyuria and the tumor. Diagnosis was reserved, but right kidney involvement strongly suspected with a bare possibility of ectopic gestation. Dr. Jones advised operation, which was concurred in, and March 5, assisted by the writer, he operated, making a long median incision. Upon palpating the tumor it became apparent that it was the greatly enlarged right kidney which was clearly multiple cystic. Examination of the left kidney showed it in a similar and almost as bad condition. All other organs appeared normal. The patient rallied nicely from the ether anesthesia, union was primary and perfect, recovery rapid and uninterrupted, the patient riding to her father's home ten miles away March 18 apparently well, except for a slight albuminuria with polyuria, voiding 50 to 65 ounces per 24 hours.

For two weeks she seemed in perfect health, was active and optimistic as formerly, and was planning to return to her own home when, at 12.40 a. m., April 1, without any premonitory symptoms, while retiring after a very happy evening spent with a few close friends at her father's home, she was suddenly stricken with a terrific headache in the vertex and frontal regions accompanied by vomiting. I saw her at 1 a. m., just before she had a slight tonic convulsion, soon to be followed by three other more severe general tonic convulsions, which were followed by a deep coma with complete relaxation of the sphincters and apparently impending death.

However, life continued, and the next 24 hours saw the sphincters resume their functions, she became conscious and recognized voices but could not see; she complained of no pain except a dull pain in her head, was very thirsty and occasionally vomited with little or no nausea. Mentally she was entirely rational, calm and even cheerful. The urine, which became very bloody, reached 80 ounces the first 24 hours it could be collected, Sp. Gr. 1006, albumin test very heavy. It also contained a gelatinous appearing substance which would not pass through filter paper. She retained nutrient enema, and within three days convalescence seemed well established and continued for fourteen days, by which time she appeared as well as formerly except for slight weakness, and the usual trace of albumin and polyuria.

April 15 she suffered another attack identical to that of April 1, only that the convulsions were even more severe, relaxing only under chloroform, and reappearing every few hours for about 24 hours. The urine was the same as before, as were all the other symptoms, only that they were more profound and did not begin to clear up so soon, still her wonderful recuperative powers asserted themselves and again she rallied, gained strength, and improved steadily until April 22, when the dreaded convulsions returned in a more mild form and again she passed into a coma which was not so deep, and there were conscious intervals of a few minutes every few hours during which intervals she received nourishment and water, recognized and conversed with relatives until a few hours before her death April 29. Her death was easy and peaceful. During the last seven days the secretion of urine gradually lessened to almost none.

The autopsy showed all the organs except kidneys perfectly normal. The right kidney on removal weighed 3 ℔ 14 oz., and measured 7 3-8x4¼x3 3-8 inches. The left weighed 3 ℔ 3 oz., and measured 6x4½x3 5-8 inches. These specimens are now in the Museum of the University of Buffalo, Medical Department.

It may be of interest to note that the father of the patient gives a history of polyuria since some years before her birth, and at the time of her death is in the advanced stage of chronic interstitial nephritis.

---

**Action of Cinchonine, Cinchonidine, and Quinidine on the Isolated Uterus.** Hale, Journal of Pharmacology and Experimental Therapeutics for May, 1915, Ther Gaz. October 1915. By adding the sulphate of these bases to oxygenated Locke's solution in which a segment of guinea-pig's uterus was suspended, it was found that all three induced strong contractions of the uterus with an increase in the strength and regularity of the spontaneous movements. These effects were still equally marked after the motor sympathetic had been paralyzed by large doses of ergot and after atropine had been given, thus indicating a direct action on the muscle. These alkaloids thus act on the uterus like quinine, but all of them are considerably more effective than quinine is. Quinidine sulphate, the most active, appears to be ten times as effective as quinine. As an explanation of this it is pointed out that quinidine is the dextrorotatory isomer of the levorotatory quinine, and thus behaves like a number of optical isomers which, having only a structural difference in chemical constitution, nevertheless differ widely in action.

---

**Urochromogen Reaction in Pulmonary Tuberculosis.** Max Biesenthal of Chicago, Ill. Med. Jour., Nov. M. Weisz, Muench. Med. Woch., 1911, found that the degree of the Ehrlich diazo test in tuberculosis varied directly as the unfavorable result but was not uniformly available. The author in 155 cases, followed the suggestion of Weisz that urochromogen, a decomposition (?) product of the normal urinary urochrome was present when the toxins of the disease were sufficient to interfere with normal oxidation. 1 C. C. of urine and 2 C. C. of distilled water are placed in each of two tubes, one being used as a control. To the other are added 3 drops of 1:1000 potassium permanganate solution. A yellowish color, varying in degree, indicates the presence of urochromogen. The amount of urochromogen present and its presence in any detectable amount is an unfavorable sign and the author believes that it is more reliable than clinical evidence, although there is an agreement in 70-80% of cases. The urochromogen test may, however, fail in moribund cases and the author warns against accepting it as absolute without much further study.

